# New Strategies in Neurogenic Heterotopic Ossification

**DOI:** 10.7759/cureus.14709

**Published:** 2021-04-27

**Authors:** Margarita-Michaela Ampadiotaki, Dimitrios S Evangelopoulos, Dimitrios Pallis, Christos Vlachos, John Vlamis, Maria-Eleftheria Evangelopoulos

**Affiliations:** 1 2nd Orthopaedic Department, KAT Hospital, Athens, GRC; 2 3rd Orthopaedic Department, KAT Hospital, National and Kapodistrian University of Athens School of Medicine, Athens, GRC; 3 3rd Department of Neurology, National and Kapodistrian University of Athens, Aiginition Hospital, Athens, GRC

**Keywords:** neurogenic heterotopic ossification, spinal cord injury, molecular treatment

## Abstract

The term neurogenic heterotopic ossification (NHO) is used to describe the pathological bone formation in soft tissues, due to spinal cord or brain injury. Commonly is presented with pain and stiffness of the affected joint. NHO affects the quality of life of these patients, delays their rehabilitation and therefore increases morbidity. The aim of this article is to emphasize pathophysiology mechanism and review new molecular treatments of heterotopic ossification (HO). It was demonstrated that potent treatment strategies are based on understanding the molecular mechanisms and aiming to inhibit the pathological process of the HO in various stages. New treatments are targeting several factors such as bone morphogenetic proteins (BMPs), retinoic acid receptors (RARs), hypoxic inhibitors (Hif1-inhibitors, rapamycin), free radical scavengers and immunological agents (imatinib). The endogenous pathways that lead to HO at molecular and cellular levels have been the aim of many studies in recent years. New treatment options for HO should be recommended due to the ineffectiveness of traditional older options, such as anti-inflammatory drugs and radiation, especially in the case of NHO.

## Introduction and background

Heterotopic ossification (HO) is defined as the extraskeletal bone formation in soft tissues, blood vessels, ligaments and muscles [1]. HO described for the first time during World War I as a complication of blast injuries and constitutes a serious reason of morbidity in soldiers. It is associated with many factors, such as trauma, burns, rare congenital diseases, brain and spinal cord injuries (SCI) [1]. Inflammatory pathways have been suggested so far to play an important role in HO. Three types of HO have been described; traumatic, neurogenic and hereditary [2].

 In this study, a thorough review on neurogenic heterotopic ossification (NHO), following SCI was performed. Although etiology still remains obscure, nowadays a correlation between triggering factors, progenitor cells’ differentiation into cartilage and bone is attempted [3,4].

In the recent years, treatment of HO is based on surgical resection following NHO maturation. However, it is pivotal to find out prophylactic treatment in order to prevent SCI-patients from HO [5]. Although nonsteroidal anti-inflammatory drugs (NSAIDs) and irradiation remain the basic prophylactic methods against HO, new molecular therapies are investigated. Furthermore, the fact that common pathophysiological mechanisms may lead to different types of HO triggers research for new molecular therapies [2].

This review provides an overview of NHO pathophysiology, conceivable treatments and possible therapeutic targets.

## Review

Epidemiology

NHO prevalence in SCI patients has been estimated to be between 10% and 53% [6]. In the study of Rawat et al., 6.3% of SCI patients developed HO, mostly at the hip joint [6]. The prevalence of NHO after SCI is lower in pediatric patients than in adults. Moreover, it has been described as spontaneous desorption of the lesions among children [7].

Risk factors

Many factors have been associated with the onset of NHO, including the duration of coma, the need for artificial ventilation and tracheostomy, and the development of vascular stasis, pneumonia or other infections. Additionally, the level of SCI is of crucial importance. Low cervical or high thoracic lesions increase the risk for NHO [8]. The disease always appears caudally to the lesion level, mainly in hip knee, elbow and shoulder joints. Other risk factors include male sex, young age and the development of autonomic dysreflexia [8,9].

Clinical presentation

NHO develops within two to four months following SCI with persistent pain, swelling, erythema, warmth and decreased range of motion. In the early stage, it is presented with localized pain, swelling and tenderness. During this phase, lesions consist of collagen fibers and fibroblasts, demonstrate high turnover and may be confused with osteosarcoma. In later stages, mature bone tissue is developed and swelling is limited. Joint range of motion is decreased affecting normal posture, leading to joint stiffness, limitation of daily activities, ankylosis and ulcerations of the skin [10].

Classification systems

Many classification systems have been proposed for HO [11]. Mavrogenis et al. suggested a classification system for NHO of the hip [12]. However, Brooker’s classification, based solely on plain radiograms, remains the most commonly used [11].

Laboratory tests and radiological images

In the early stages, alkaline phosphatase (ALP) increases with a pick at 10 weeks. Levels of calcium, phosphorous, C-reactive protein (CRP), erythrocyte sedimentation rate (ESR), Creatine phosphokinase (CPK), matrix metalloproteinase 9 (MMP9) and IL-3 have been suggested as follow-up markers; however, their use remains controversial [13].

**Figure 1:**
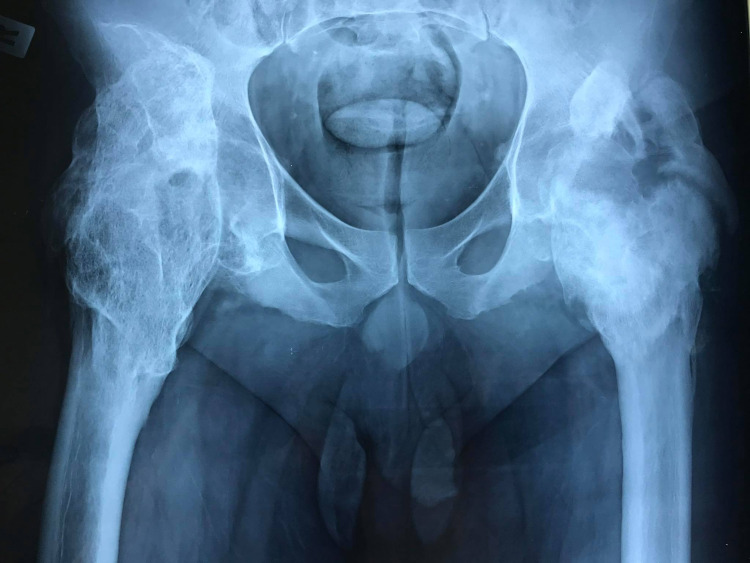
Anteroposterior X-ray of a 38-year-old paraplegic patient showing neurogenic heterotopic ossification in both hips following T1 fracture.

Plain X-ray is the initial imaging study for HO diagnostic algorithm (Figure 1).

Nuclear bone scans and ultrasonography represent useful early diagnostic tools, specifically for patients following SCI while computed tomography (CT) and magnetic resonance imaging (MRI) are applied mainly for the diagnosis of mature HO [14,15]. The three-phase radionuclide bone scan still remains the “gold standard” for early HO detection [16].

Pathogenesis of HO

Neuroinflammation

SCI initiates a cascade of pathophysiological changes that lead to heterotopic bone formation. Especially, neuroinflammatory substances such as oncostatin M (OSM), glutamate, substance P, calcitonin gene-related protein (CGRP), transforming growth factor beta (TGF-β) and leptin are produced from the injured spinal cord and activate immune cells such as macrophages [17]. In addition, lesions of the spinal cord damage blood barrier integrity and permit transfer of additional inflammatory molecules [18].

In the early stage, local inflammation, as a response to spinal cord and peripheral tissues’ injury (muscle, fracture, etc.), initiates a cellular and molecular cascade [5]. During this stage, inflammatory cells, such as macrophages, lymphocytes and mast cells are gathered in the perivascular area of HO lesions, triggering progenitor cells’ proliferation [19].

Bone tissue develops from progenitor cells, through endochondral and intramembranous pathways. HO commonly occurs by endochondral ossification [20]. Tissue-derived mesenchymal stem cells (MSCs) participate in bone healing and heterotopic bone formation by differentiating into osteoblasts, chondrocytes or adipocytes. Local microenvironment has been shown to affect these pathways [21]. Injury-induced hypoxia activates HIF1a, a protein which triggers the differentiation of progenitor cells into osteoblasts and chondrocells. Current studies suggest that mesenchymal progenitor cells (MPCs) of traumatically injured muscle actively participate in HO formation [19]. Furthermore, cells such as pericytes, vascular endothelial cells, skeletal muscle cells, hematopoietic cells, mast cells and MPCs have osteogenic abilities and participate in HO formation [4].

Although local inflammation leads to the expansion and differentiation of multiple cells, the exact pathway for HO remains yet unclear. The differentiation of progenitor cells towards a chondrogenic lineage and endochondral ossification depends on factors, such as BMP signaling, SOX and hypoxic conditions [20]. On the other hand, differentiation towards osteogenesis and bone formation is supported through vascularization, Wnt/β-catenin signaling and nuclear factors (Runx2 and Osterix) [21]. Kan et al reported a common mechanism for all types of HO [4]. Furthermore, current studies present the important role of BMP, Hedgehog (HH), Wnt/β-catenin, FGF, and HIF-1α [22] .

Bone Morphogenetic Protein (BMP) signaling

BMPs belong to the family of transforming growth factor β (TGFβ). BMPs-induced bone formation occurs under pathological procedures. Expression of BMP-2, 4, 7 and 9 was upregulated in animal models with SCI-induced HO. BMP-9 is also implicated in HO pathophysiology and depends on muscles’ microenvironment [23]. Studies on fibrodysplasia ossificans progressiva (FOP) and traumatic brain injury (TBI) patients report potent BMP signaling [18].

Hedgehog Signaling

The Hedgehog (HH) signaling pathway induces chondrocyte and osteoblast lineage differentiation. HH is implicated in osteogenesis during fracture-healing through regulation of angiogenesis [24]. Absence of GNAS leads to HH signaling increase, in mesenchymal progenitor cells. Moreover, the literature supports that HH inhibitors may prevent progressive osseous heteroplasia (POH) and other forms of HO [24].

HIF1a (Hypoxia-Inducible Factor-1α)

NHO is caused by hypoxia-induced tissue damage [25]. Tissue ischemia, due to vascular system damage, leads to immune cell response and cell proliferation. Hypoxic environments induce chondrogenic cell differentiation. HIF-1α is a regulator of cellular hypoxic responses [25]. HIF-1a is significantly up-regulated during chondrogenic differentiation, in contrast to decreasing levels in the osteogenic stage [26]. Oxygen deficiency inhibits cell growth and increases apoptosis. Furthermore, the stability of HIF-1α increases gene-transcription, enhancing adaptation to hypoxia. Studies have shown that hypoxia, through HIF1a, affects the production of BMP, VEGF and cytokines, increases chondrogenic cells proliferation and promotes ectopic bone formation [27]. Wang et al. presented the correlation between inflammation and hypoxia [26]. The authors stated that cellular hypoxia enhances heterotopic endochondral ossification by exaggerating BMP-signaling through rabaptin 5 (RABEP1)-mediated retention of activin A receptor type I (ACVR1). Activin A probably plays an important role in NHO, since high levels of this substance have been detected in the serum of these patients [27]. Agarwal et al. stated that HIF-1α is crucial for all forms of HO [28].

Mammalian Target of Rapamycin (mTOR)

The mTOR signaling not only enhances the angiogenetic role of HIF1a but also activates vascular endothelial growth factor VEGF. In addition, mTOR signaling pathway is implicated in chondrogenic differentiation. Studies on FOP patients showed that mutation of ACVR1 receptor and abnormal Activin levels lead to increased mTOR signaling [29].

Retinoic Acid Receptor (RAR) Signaling

There are three types of RARs; RARα, RARβ, RARγ, which are inhibitors of chondrogenesis and cartilage formation. It has been found that only selective agonists of RARγ are effective in preventing HO [30]. Recent literature data support their ability to block the consolidation of skeletal progenitor cells and their differentiation into chondrocytes [30].

Treatment options 

Conventional Treatments

(i) NSAIDs (nonsteroidal anti-inflammatory drugs): Patients with NHO are presented with severe systemic inflammation. NSAIDs have been widely used for HO prevention and treatment. Ketorolac, ibuprofen, celecoxib and indomethacin are commonly used to prevent HO in patients after total hip arthroplasty. In trauma patients, their efficacy has not yet been proved [31]. Polytrauma patients suffer from additional injuries that may be negatively affected by the NSAIDs. Administration of NSAIDs following acute trauma may induce bleeding, gastritis and impair fracture healing. Nevertheless, a recent study on SCI patients showed that the use of NSAIDs during early injury stages may prevent HO development [31].

(ii) Radiation therapy: Radiotherapy affects mesenchymal cells, responsible for HO formation [32]. Single-dose radiation therapy demonstrates good results on SCI-induced HO treatment. However, long-term side effects still remain unknown [32]. Cipriano et al, in a case-control study on NHO patients treated with radiation therapy, pointed out that the incidence of HO was higher in the treatment group (15.0%) compared to the control group (5.1%) [33].

Novel Therapies

(i) Biphosphonates: Biphosphonates promote osteoclasts’ death and reduce calcification. Etidronate inhibits bone mineralization; however, organic matrix remains unaffected and bone formation reoccurs, if treatment continues for 6 months, following injury [34]. Furthermore, SCI patients, with no-visible lesions on CT-scan treated with etidronate, had a significantly lower incidence of NHO, compared to patients with radiographically detectable NHO. If bone mineralization has already occurred, bisphosphonates are ineffective [34].

(ii) Inhibitors of BMPs: BMP-signaling is crucial for skeletal growth and HO formation [10]. The inhibitors of BMP; Noggin-glycoprotein- and BMP type 1 receptor inhibitors inhibit dimerization of BMP receptor and Smad-dependent signaling [10]. These inhibitors are effective in all stages of HO. However, side effects though BMP receptor inactivation have been described in multiple organs.

Noggin belongs to TGF-β proteins and is implicated on skeletal growth [35]. Hannallah et al. studied Noggin in muscle-derived stem cells, in a range of doses. They suggested that the development of heterotopic ossification is inhibited by the block of BMPs [35]. Glaser et al stated that the local concentration of wild-type Noggin or the somatic cell gene transfer of a Noggin mutation can inhibit HO [36]. Overexpression of Noggin prompts osteopenia reduces bone mineralization and density. Additionally, it affects other organs, such as the pituitary, heart, prostate, thymus and parathyroid. Animals treated with Noggin showed no HO signs on X-rays, thus proving that gene therapy with Noggin is a promising method for HO treatment [37].

Transforming growth factor beta (TGF-β) has also been suggested as therapeutic option for HO. For HO inhibition, it may be advantageous to decrease the amount of phosphorus, essential for ATP and ADP increase. The differentiation of MSCs into osteochondral cells occurs through SMAD phosphorylation. In animal models, apyrase, a SMAD1/5/8 inhibitor, decreased the amount of HO [37]. Yu et al suggested that small molecule inhibition of BMP type I receptor could be beneficial in the treatment of FOP and heterotopic ossification associated with excessive BMP signaling [38]. In recent studies, minor molecule inhibition of TGF-β activates kinase-1 and reduces side effects, such as weight loss, delayed wound healing and infection [39].

(iii) Retinoic acid agonists-RARγ: Of the three known types of retinoic acid receptors (RAR); α, β, γ known to prevent chondrogenesis, only retinoic acid receptor gamma (RARy) agonists are used for HO [13]. Retinoid acid prevents the differentiation of chondrogenic precursor cells into chondrocytes, in the early stages of HO. Palovarotene, a RAR-y agonist, already known from the clinical trial of α-1-antitrypsin-induced emphysema is known to prevent the development of HO [40-43]. Moreover, the use of Palovarotene on patients with hereditary multiple exostoses resulted in a 50% decrease of HO [19]. However, Palovarotene is teratogenic and prompts limb malformations in immature skeletons. Additionally, it may lead to pancreatitis, vision impairment, mucocutaneous ulcers and sensitivity to sunlight [44]. Μore studies are required to evaluate RARγ efficacy, due to its wide expression, on chondrocytes and chondrogenic cells, compared to RARa and RARß [13].

(iv) Rapamycin- Inhibitor of hypoxia-inducible factor 1a-( HIF1a): It is well-known that hypoxia-inducible factor-1α (Hif1α) orchestrates cellular adaptation to hypoxia. Hif1a is expressed in trauma-induced mouse models. BMP pathway is also enhanced by hypoxia. Literature data supports that the use of rapamycin, an Hif1a inhibitor, prevented HO formation in animal models [45]. Rapamycin is a macrolide immunosuppressant, which inhibits the mechanistic target of rapamycin (mTOR) protein kinase [45,46]. It blocks the mTOR pathway, preventing HIF-1α translation without affecting its transcription. Rapamycin also inhibits the hypoxia-induced expression of VEGF [44, 45]. Despite its wide application on patients following kidney and liver transplant, its side effects are well-known, including hyperlipidemia, hypercholesterolemia, hypertriglyceridemia, glucose intolerance, insulin resistance and diabetes, anemia, thrombocytopenia, dermatological and gastrointestinal disorders, sinusitis, respiratory and urinary infections, and testicular dysfunction [45]. Maekawa et al. studied the use of rapamycin on ACVR1 mutant mice and pointed out that this macrolide decreased the occurrence of HO and reduced the amount of recurrent HO following surgical resection [46]. Recent data shows that, in knock-out HIF-1α mice, HO progenitor cells are diminished [47].

(v) Free radical scavengers: The ischemia-reperfusion syndrome and the disuse phenomenon are the main causes of production of free radicals. Hypoxia-induced free radicals prompt oxidative damage to the cells and induce HO formation. Free radical scavengers such as allopurinol and N- acetylcysteine have been suggested as inhibitors of HO. An experiment in rodents pointed out that FR scavengers are effective in preventing HO. Their effectiveness was assessed as more potent than that of indomethacin [48].

(vi) Immune therapy-Imatinib: Ιmatinib is indicated generally for chronic myeloid leukemia. It is considered to be a safe medication with only mild side effects, such as: cramps, diarrhea, edema and skin rashes [49]. Platelet-derived growth factor (PDGF), responsible for neoangiogenesis at the final stages of endochondral ossification, is believed to participate in the HO formation [50]. It has been shown that imatinib is a potent PDGF inhibitor in murine models. The amount of HO was decreased up to 85%, following the administration of imatinib. Other animal studies also demonstrated that imatinib blocks HIF1a and affects HO progression [24]. Furthermore, imatinib inhibits macrophages and mast cell proliferation as well as the excretion of metalloproteases. Nevertheless, it remains unknown how imatinib affects wound healing [50]. Kaplan et al., in the only study on human subjects, reported a small case series on seven children with FOP and pointed out the hopeful use of imatinib in HO prevention [49]. It is imperative to perform additional clinical trials to understand the profits of imatinib in the treatment of HO (Table 1).

**Table 1:** Molecular treatments and their efficacy. FOP: fibrodysplasia ossificans progressiva; HO: heterotopic ossification; NHO: neurogenic heterotopic ossification; RAR: retinoic acid receptors; BMP: bone morphogenetic proteins; HIF1a: hypoxia-inducible factor-1α.

Author	Treatment	Model	Results
Chakkalakal et al. [40]	Palovarotene	Mouse FOP trauma induced	80% reduction of HO
Genet et al. [3]	Prophylactic depletion of macrophages by clodronate-loaded liposomes	Transection T7-T8 and cardiotoxin injection in mice	90% reduction of NHO
Pavey et al. [13]	Palovarotene	110 rats blast injured	46%-100% reduction of HO
Shimono et al. [41]	RAR agonist	Mouse BMP injected	Reduction of the ratio mineralized tissue volume/total volume histologically
Wheatley et al. [42]	Palovarotene	Blast-related amputation-72 rodents	Reduction of cytokines
Agarwal et al. [28]	Rapamycin	Mice, burned and genetically modified	Reduction of de novo HO formation
Hannallah et al. [35]	Noggin	Achilles tenontotomy in mice	83% reduction of HO
Lees-Shepard et al. [19]	Palovarotene	Mouse genetic model	50% reduction of HO
Maekawa et al. [46]	Rapamycin	Mice with mutation of ACVR/ALK2 gene	75% reduction of HO formation
Qureshi et al. [45]	Rapamycin	Mice trauma induced	90.3% reduction
Glaser et al. [36]	Noggin	BMP4-induced mouse model	Noggin inhibited HO
Vanden Bossche et al. [48]	Free radical scavengers [allopurinol and N-acetylcysteine]	Rabbits	Higher inhibitory effect than indomethacin
Zimmermann et al. [47]	Echinomycin (HIF1a inhibitor)	Murine model-Achilles tenotomy	90% reduction
Yu et al. [38]	Selective inhibitor of BMP type I receptor kinases, LDN-193189	Mouse FOP model	Reduction of HO
Peterson et al. [37]	Apyrase	Burn-Achilles tenotomy mice	Decrease HO formation
Werner et al. [50]	Imatinib	Murine model Achilles tenotomy	85% reduction of HO

## Conclusions

This article is a review of the current literature on neurogenic heterotopic ossification. Limitation of this review is the small samples of studies on NHO. Traditional management of heterotopic ossification included NSAIDs, radiation therapy and surgical excision. However, since traditional therapies are not always effective, recent studies focus on pathophysiologic pathways to develop new molecular therapies. As neurogenic heterotopic ossification remains a major cause of disability and morbidity for SCI patients, it is imperative to perform more studies to clarify the safety and the effectiveness of these therapies.
